# Herbicide Leaching in Soil with Different Properties: Perspectives from Commercial Formulations and Analytical Standards

**DOI:** 10.3390/toxics12030199

**Published:** 2024-03-02

**Authors:** Rita de Cássia Araújo de Medeiros, Tatiane Severo Silva, Taliane Maria da Silva Teófilo, Francisca Daniele da Silva, Matheus de Freitas Souza, Ana Beatriz Rocha de Jesus Passos, Bruno Caio Chaves Fernandes, Hamurábi Anizio Lins, Paulo Sergio Fernandes das Chagas, Carolina Malala Martins Souza, Ioná Santos Araújo Holanda, Daniel Valadão Silva

**Affiliations:** 1 Department of Agronomic and Forestry Sciences, Plant Science Center, Universidade Federal Rural do Semi-Árido—UFERSA, Av. Francisco Mota, 572, Costa e Silva, P.O. Box 137, Mossoró 59625-900, RN, Braziltalianeteofilo23@gmail.com (T.M.d.S.T.);; 2 Department of Plant and Agroecosystem Sciences, University of Wisconsin, Madison, 1575 Linden Dr., Madison, WI 53706, USA; severosilva@wisc.edu (T.S.S.); hamurabi_a_@hotmail.com (H.A.L.); paulosfc@ufersa.edu.br (P.S.F.d.C.); 3 Department of Agronomy, Universidade de Rio Verde, Rio Verde 75901-970, GO, Brazil

**Keywords:** herbicide leaching, herbicide–soil interaction, herbicide behavior

## Abstract

The leaching of herbicides into the soil is essential to control germinating seeds and parts of vegetative weeds. However, herbicide transportation to deeper soil layers can result in groundwater contamination and, consequently, environmental issues. In this research, our objective was to investigate differences in herbicide leaching between commercial formulations and analytical standards using three different soils. Leaching experiments were carried out for diuron, hexazinone, and sulfometuron-methyl herbicides isolated and in binary and ternary mixtures. The herbicide residue quantification was performed by ultra-high-performance liquid chromatography coupled to a mass spectrometer (LC-MS/MS). Diuron had less mobility in soils and was retained in the most superficial layers. Hexazinone and sulfometuron-methyl were more mobile and leached into deeper layers. The leaching process was more intense for hexazinone and sulfometuron-methyl. The additives present in the commercial formulation favored the leaching in soils of diuron, hexazinone, and sulfometuron-methyl herbicides isolated and mixture compared to the analytical standard. This fact highlights the importance of considering these effects for the positioning of herbicides in the field to increase the efficiency of weed control and minimize the potential for environmental contamination.

## 1. Introduction

The vertical transport of herbicides in the soil profile, a phenomenon known as leaching, is essential for incorporating products applied in pre-emergence to control weeds in agricultural areas as they emerge. The intensity of herbicide leaching into soils depends on factors such as the application method, the environmental conditions, and the properties of the herbicide molecule [1,2,3]. The interaction between these factors will directly influence the distribution of the herbicide in the soil profile and, consequently, its efficiency in controlling weeds and the potential for environmental contamination [4,5].

The distribution of weed propagules in the soil varies at different depths, requiring different control strategies. Mixing herbicides has been employed as a tool to enhance the efficiency of commercial formulations in weed control. This is achieved by broadening the spectrum of action through the addition of multiple herbicide molecules [6]. For instance, in Brazil, a commercial formulation combining the herbicides diuron and hexazinone is used. The first herbicide aims to control weeds germinating near the soil surface due to its limited mobility, while the second one also reaches deeper layers, effectively controlling weeds germinating further down in the soil [7]. In recent years, a triple mixture containing the herbicides diuron, hexazinone, and sulfometuron-methyl has been developed for application in sugarcane cultivation [7,8]. When isolated, these herbicides have an average field half-life of 90, 90, and 28 days, respectively, and are associated with a moderate to high risk of potential leaching [9,10].

Although the use of herbicides in mixed formulations increases the likelihood of weed control [11], a previous study showed that the sorption in the soil of the herbicides diuron, hexazinone, and sulfometuron-methyl is reduced when used in a mixture, which would cause a more significant loss of herbicides in the soil profile and would increase the risk of contamination of ground and surface water [5]. The authors considered only the active ingredient in this study, disregarding the commercial formulation (active ingredients and additives). In an aquatic risk assessment that estimated the potential risk of individual herbicides and co-acting herbicides in herbicide mixtures in South Florida freshwater ecosystems, it was found that herbicide mixtures (primarily with diuron) posed a high aggregate ecological risk to Lee, Martin, and St. Lucie counties in South Florida [12].

Most additives present in herbicide formulations have surface-active properties and, in many cases, can result in decreased pesticide sorption and increased environmental mobility [13]. Leaching of the commercial formulation of the pesticides azoxystrobin, cyproconazole, propyzamide, and triadimenol was observed to be more pronounced in the mixture and occurred earlier compared to treatment with the analytical standard of these pesticides [4]. This led to the assumption that additives might influence the sorption of the pesticides’ active ingredients by the soil constituents. Furthermore, the extent of this effect can be expected to vary between single and mixed products.

The influence of the pesticide formulation on its behavior is also dependent on the type of soil [14]. For instance, Földényi et al. [13] observed that the amount of the dispersant Supragil WP adsorbed correlated with the total organic carbon content and specific surface area of the studied soils. Additives can assist in peptizing soil organic matter and stabilizing the solution phase by hindering the adsorption (depending on soil pH) of the active ingredient, thus increasing the mobility of pesticide molecules in the soil [13,15].

It is expected that the environmental fate (e.g., soil herbicide leaching) of the active ingredients can be altered by the additives present in the herbicide formulations. The novelty presented by our study is its investigation into the effect of a commercial formulation, which contains multiple active ingredients, on herbicide leaching. This emphasis underscores the importance of considering the complete herbicide formulation (including both active ingredients and additives) when assessing the fate of herbicides in the environment. The comparison between commercial formulation and analytical standards will make available relevant potential leaching data based on soil columns under laboratory conditions that can additionally provide an estimate outcome of potential formulated herbicide leaching to groundwater. The experiment aims to evaluate whether the commercial formulation intensifies the leaching of diuron, hexazinone, and sulfometuron-methyl when applied in isolation and as a mixture, compared to the analytical pattern of these herbicides in soils with different attributes.

## 2. Materials and Methods

### 2.1. Soil Characterization

Soil samples with contrasting properties were collected from three different locations: LV (Mossoró, Rio Grande do Norte, 5.06 S, 37.40 W); RQ (Pedro Velho, Rio Grande do Norte, 6.43 S, 35.22 W), and CX (Quixeré, Ceará, 5.07 S, 37.80 W). The soils were collected from the superficial layer of 0–20 cm, at sites with no history of herbicide application. To confirm the absence of herbicides in the collected soils, soil samples were fortified with a known concentration of the herbicide and then compared with unfortified samples. The absence of herbicides in the soils was demonstrated using chromatograms. Subsequently, the soils were air-dried, sieved through 2 mm meshes, and characterized (chemically and physically) as shown in Table 1 [16].

**Figure 1:**
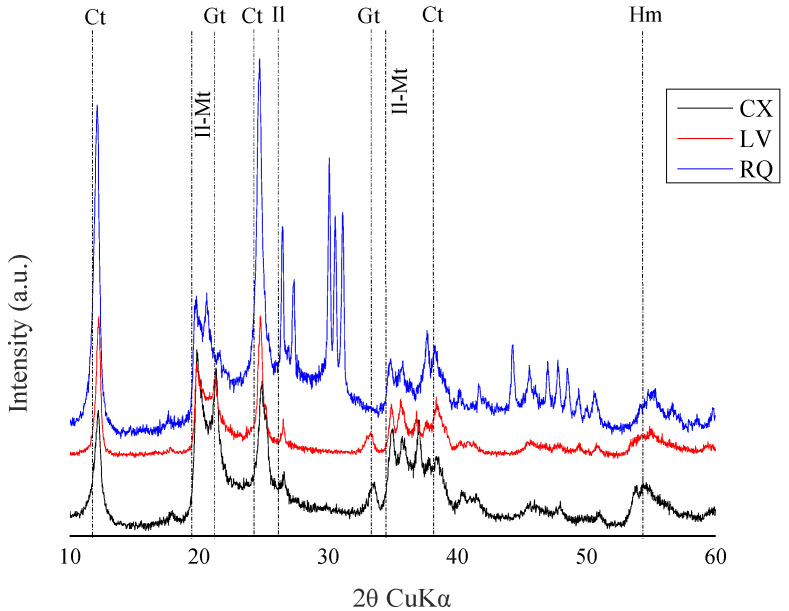
X-ray diffractometry of the clay fraction from the A horizons of Haplic Cambisol (CX), Red Latosol (LV), and Quartzarenic Neosol (RQ). Ct: Kaolinite; Il: Illite; Mt: Montmorillonite; Gt: Goethite; Hm: Hematite; Gb: Gibbsite; and Qz: Quartz.

Mineralogical content was assessed, given its significant influence on the behavior of pesticides in the soil [18]. The mineralogical characterization of the soils was conducted using X-ray diffraction (XRD) at the Integrated Center for Technological Innovation in the Semi-Arid Region (CITED)—UFERSA (Figure 1). The identification of minerals was performed in the clay fraction through chemical dispersion with 0.025 mol L^−1^ of sodium hexametaphosphate and mechanical dispersion using a “Wagner”-type stirrer for 16 h [17]. The XRD test was performed using a diffractometer (model XRD—6000, Shimadzu Corporation, Kyoto, Japan), with copper kα1 emission, operating at a 40 kV source potential and a 30 mA current. The sweep speed had a step of 0.02° per second, and the scanning range ranged from 5 to 65° (2θ scale). Qualitative interpretations of the spectrum and identification of mineral peaks were performed with the assistance of the Raio X v program 1.0.0.37, and the phases were identified following the methodology of Chen [19].

### 2.2. Experimental Design

The experiments were conducted at the Weed Management Laboratory of the Federal Rural University of Semi-Árido, Mossoró, RN, Brazil. The experiment was conducted as 2 × 3 factorial in a completely randomized design with three replications. The first factor was the herbicide formulation (commercial and standard), and the second factor corresponded to the isolated herbicide and its binary and ternary mixtures. The leaching studies were conducted on polyvinyl chloride (PVC) columns using three soils (Red Latosol—LV; Quartzarenic Neosol—RQ; and Haplic Cambisol—CX) and seven depths (0–5, 5–10, 10–15, 15–20, 20–25, 25–30, and 30–35 cm). The evaluations were carried out in triplicate using the ultra-efficiency liquid chromatography technique coupled to a mass spectrometer (LC-MS/MS) following the guidelines of the OECD—312 Leaching in Soil Columns [20].

### 2.3. Chemical Products

The herbicide standard of diuron [3-(3,4-dichlorophenyl)-1,1-dimethylurea] and hexazinone [3-cyclohexyl-6-dimethylamino-1-methyl-1,3,5-triazine-2,4 (1H, 3H)-dione] were purchased from Sigma-Aldrich^®^ (Saint Louis, MO, USA) with chemical purities of 99.6 and 99.5%, respectively. Sulfometuron-methyl {methyl 2-[(4,6-dimethylpyrimidin-2-yl) carbamoyl sulfamoyl] benzoate} was obtained from the company Supelco (Bellefonte, PA, USA) with a purity of 99.5%. Stock solutions of each herbicide were prepared at a concentration of 1000 mg L^−1^ in acetonitrile. Standard working solutions were made from the stock solution of each herbicide in distilled water. The herbicides with commercial formulations used were Diox^®^ (500 g L^−1^ a.i.) (Ouro Fino Química LTDA); Hexazinone^®^ (250 g L^−1^ i.a.) (Nortox S/A); Curavial^®^ (750 g L^−1^ a.i.); and Front^®^ (603 + 170 + 14.5 g L^−1^ a.i.) (Du Pont do Brasil S/A). The reagents used were all analytical- and HPLC-grade.

### 2.4. Preparation of Columns

**Figure 2:**
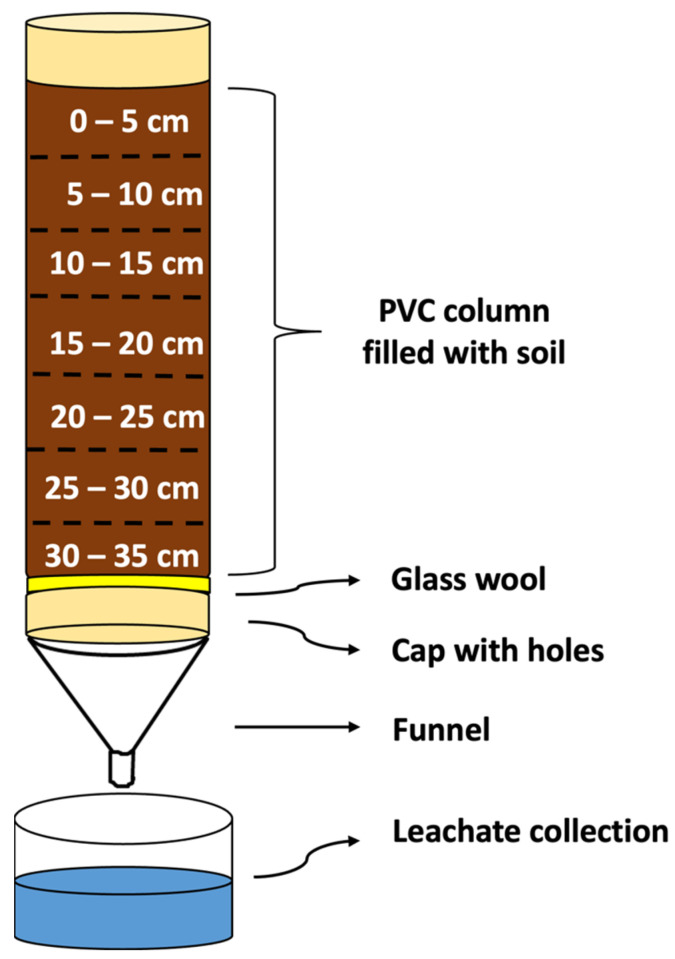
Pictorial diagram of the leaching column setup fabricated for the experiment.

The experimental units consisted of columns made of an inert material based on PVC (10 cm in diameter × 35 cm in length), filled with the three soils (4 kg) individually. The columns were previously waterproofed with paraffin to prevent lateral water flow, thus ensuring uniformity in the water flow. A glass wool disc (2 cm) was placed at the base of each column to retain the soil and allow drainage. The bases of the columns were sealed with a PVC plug, with holes at the bottom that allowed water drainage and leachate collection. Initially, the columns were placed vertically in a water tank until complete capillary saturation. Then, they were placed in support racks, allowing the water to flow for 24 h so that the soil moisture was close to the field capacity. A pictorial diagram of the leaching column setup fabricated for the experiment is shown in Figure 2.

### 2.5. Herbicide Application

Diuron, hexazinone, and sulfometuron-methyl solutions were prepared according to the highest recommended commercial dose (0.73, 0.45, and 0.8 mg L^−1^, respectively) for sugarcane cultivation. Each column of soil was treated individually, receiving 10 mL of herbicide solution on its surface. The solution was evenly distributed using a beaker. After 24 h of application, a rainfall of 60 mm was simulated over a four-hour period (15 mm of rain applied every hour) onto the soil columns. The leachate from the columns was collected and stored at −20 °C until quantification.

After collecting the leachate, the columns were left to rest for 72 h. Then, they were placed horizontally for soil removal and sectioning, with samples taken every 5 cm in depth (0–5; 5–10; 10–15; 15–20; 20–25; 25–30; 30–35 cm). Following air-drying, the soil samples were crushed to break up clods, sieved through a 2 mm mesh, and stored at −20 °C. LC-MS/MS was used to analyze each segment of soil and leachate for herbicide quantification.

### 2.6. Herbicide Extraction

For the analysis of diuron, hexazinone, and sulfometuron-methyl in leachate samples, a 1 mL aliquot was placed into 1.5 mL microtubes. Subsequently, the microtubes were centrifuged at 2260× *g* for five minutes. Using a syringe, the entire sample was then removed and filtered through a polyvinylidene fluoride (PVDF) membrane with a pore size of 0.22 µm and transferred to vials for further analysis by LC-MS/MS.

The QueChERS method, as described by Pang et al. [21], was applied for herbicide extraction with modification and validation. A solution of 10.0 mL of acetonitrile, 100 µL of acetic acid, and 2.0 mL of distilled water was added to Falcon tubes containing 5 g of soil. The tubes were then placed in an ultrasound bath for 15 min. Subsequently, 1.0 g of NaCl and 2.0 g of MgSO_4_ were added to each Falcon tube. The samples were vortexed at 25 °C ± 2 °C until the solution was homogenized. After shaking, the samples were centrifuged at 2260× *g* for five minutes. A 1 mL aliquot was collected from the supernatant in the Falcon tubes and placed directly into 1.5 mL vials containing 0.2 g of magnesium sulfate (MgSO_4_). The vials were vortexed for an additional five minutes. The supernatant was then transferred into a syringe with a 0.22 µm Nylon membrane and further transferred to vials for herbicide quantification using LC-MS/MS.

#### 2.6.1. Extraction Method Validation

The validation of the modified QueChERS method considered the following parameters: linearity, selectivity, the limit of quantification (LOQ), the limit of detection (LOD), accuracy (recovery), and precision (repeatability and intermediate precision) by the guidelines of the Brazilian Health Regulatory Agency (ANVISA): https://www.gov.br/anvisa/pt-br, accessed on 14 July 2019 [22].

The linearity of the diuron, hexazinone, and sulfometuron-methyl extraction method was determined from the analytical curves obtained by injecting standard solutions (n = 7) at seven different concentrations of the analytes (0.5, 1.0, 2.5, 5.0, 10, 25, and 50 µg kg^−1^), prepared by successive dilutions of the working solution. The linear correlation coefficient (R^2^) was evaluated from these data, thereby determining the method’s linearity (Table 2). Method selectivity was assessed by comparing the chromatograms of blank extracts and fortified samples at a concentration of 10 µg kg^−1^ in the soil matrix.

The method’s limits of detection (LODs) and quantification (LOQs) were determined using solutions at concentrations that were 3 and 10 times the standard deviation ratio of the linear regression coefficient with the angular coefficient of the analytical curve, respectively. The precision of the method was evaluated through repeatability, which was tested consecutively at three levels of fortification (1, 10, and 100 mg L^−1^) and expressed as the relative standard deviation (RSD) (Table 3).

#### 2.6.2. Recovery Study

The recovery test of diuron, hexazinone, and sulfometuron-methyl in soils was conducted using analyte concentrations of 1, 10, and 100 µg kg^−1^, with the analysis performed in triplicate. The samples were concentrated in 50 mL Falcon tubes containing 5.00 g of soil and 1 mL of the incorporation solution. These samples were stored in the absence of light until the solvent completely evaporated. The control treatment (not fortified) was maintained under the same conditions for comparison with the results of the fortified samples and the quantification of the recovered herbicides (Table 3).

### 2.7. Chromatographic Conditions

The chromatographic conditions used in this study consisted of the use of an ultra-high-performance liquid chromatography (UHPLC) system coupled to a triple quadrupole mass spectrometer (LCMS-8040, Shimadzu, Tokyo, Japan) (liquid chromatography/tandem mass spectroscopy—LC-MS/MS). The column used in the UHPLC was a Restek (Pinnacle DB AQ C18, size 50 × 2.1 mm, with 1.9 µm particles), including two LC—30AD pumps, a DGU degasser—20A_5R_, a Sil autosampler—30AC, a CTO—30AC column oven, and a CBM−20^a^ controller.

The chromatographic system was operated with isocratic elution using a flow rate of 0.3 mL/min, an injection volume of 5 μL, and an automatic sampler temperature of 15 °C. Mobile phase A consisted of water with 0.1% formic acid, while mobile phase B was acetonitrile in a 30-to-70% proportion, with a column oven temperature of 40 °C.

The mass spectrometer was operated using positive ionization with multiple reaction monitoring (MRM). Two transitions of each pesticide analyzed were monitored, and the most stable transition was chosen for quantification, while the second was used to confirm the results (Table 4).

**Figure 3:**
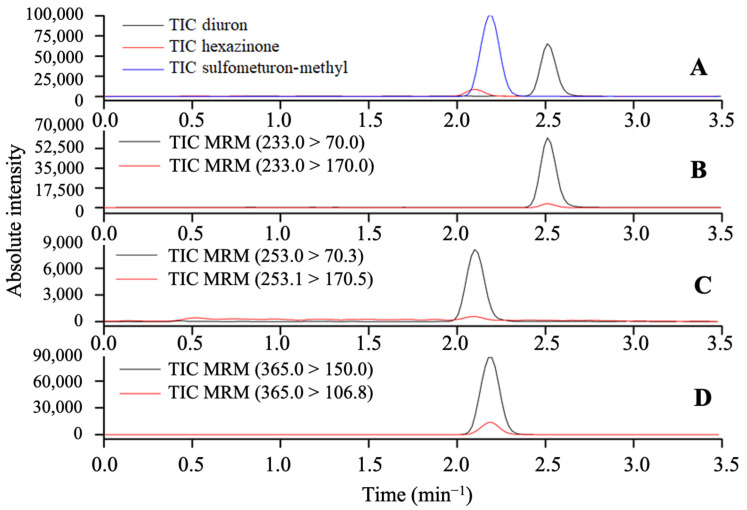
MRM mode, total ion intensity chromatogram for the herbicides diuron, hexazinone, and sulfometuron-methyl (**A**), intensity of the daughter ions of diuron (**B**), hexazinone (**C**), and sulfumeturon-methyl (**D**).

The following parameters were used for the LC-MS/MS analysis: interface voltage of 4.5 kV, with a desolvation line temperature of 250 °C and a nebulizing nitrogen gas flow of 3 L min^−1^; a block temperature of 400 °C; a drying nitrogen gas flow at 15 L min^−1^; and collision argon gas with a pressure of 230 kPa. The selectivity of the method was confirmed by considering the MRN transitions (*m*/*z*) and the absence of signals from interfering matrix compounds at the same retention time as diuron, hexazinone, and sulfometuron-methyl (Figure 3). 

### 2.8. Statistical Analyses

Data were submitted to the Shapiro–Wilk residual normality test [23] and homoscedasticity [24]. Once normality and homoscedasticity were confirmed, an ANOVA was performed using the F test (*p* ≤ 0.05), and for significant results, means were compared using the Tukey test (*p* ≤ 0.05). All figures were generated with mean values (n = 3) accompanied by the mean confidence interval (*p* ≤ 0.05) using the RStudio software (version 3.6.3, Team R Core) [25].

## 3. Results and Discussion

Diuron, hexazinone, sulfometuron-methyl, and their mixtures, including both the commercial and standard formulations, were not detected in the leachate water of the LV, CX, and RQ soils. Therefore, the focus of the results will be on herbicide leaching from soil depths D1 (0–5 cm) to D7 (30–35 cm).

### 3.1. Leaching of Commercial Formulations and Diuron Analytical Standard

#### 3.1.1. Red Latosol (LV)

**Figure 4:**
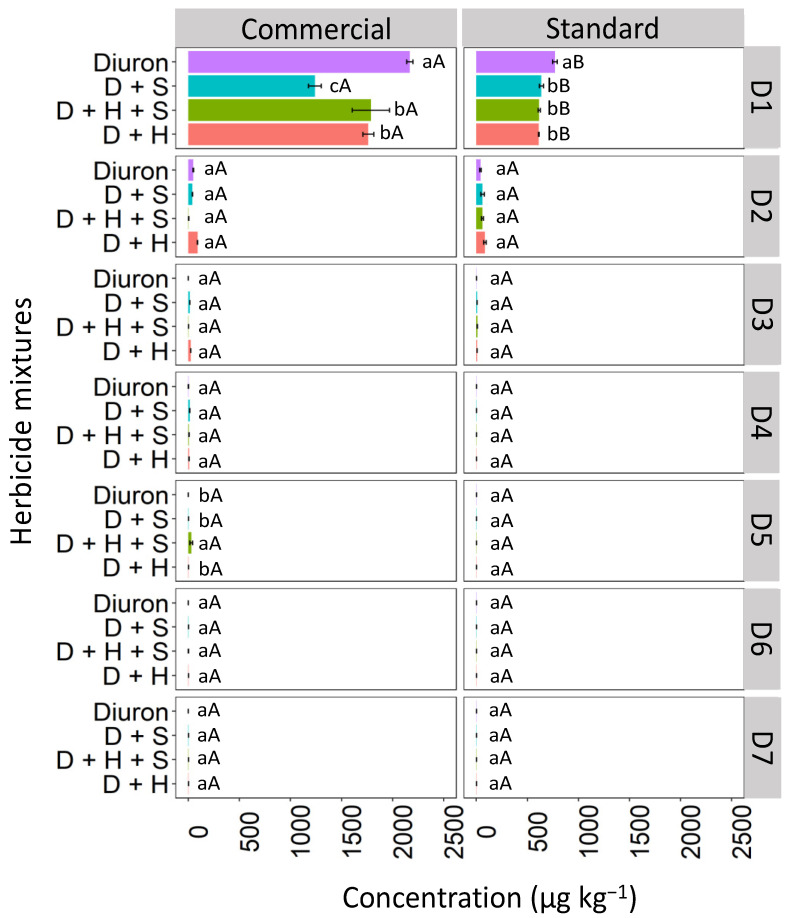
Leaching (μg kg^−1^) of diuron isolated and in binary and ternary mixtures for commercial and standard formulation in Red Latosol. Lowercase letters compare means between mixtures, and uppercase letters compare formulations (commercial and standard). Bars indicate the standard error of the mean (n = 3). D + S (diuron + hexazinone), D + H + S (diuron + hexazinone + sulfometuron-methyl), D + H (diuron + hexazinone). D1 to D7 represent the soil depths.

Isolated diuron exhibited a higher concentration at the first soil depth (D1: 0–5 cm) when compared to the binary and ternary mixtures in both commercial and standard formulations (Figure 4). Diuron, when isolated, and in the commercial formulation, showed higher concentrations in the D1 layer compared to the standard formulation. In the commercial formulation, isolated diuron showed a higher concentration at depth D1 (2253.6 μg kg^−1^) compared to the mixtures. The commercial mixtures of diuron + hexazinone + sulfometuron-methyl (D + H + S) and diuron + hexazinone (D + H) displayed similar behavior, with reductions in concentration of 13.76% and 13.91%, respectively, compared to isolated diuron. The diuron + sulfometuron-methyl (D + S) mixture exhibited a significant difference from the other treatments, with a 44.75% reduction in concentration compared to isolated diuron. In the standard formulation, a higher concentration of diuron was isolated at depth D1 (754.6 μg kg^−1^) compared to diuron applied in binary and ternary mixtures, which did not differ from each other. These mixtures showed an approximately 13% reduction in concentration compared to the isolated treatment. From depth D2 (5–10 cm) to D7 (30–35 cm), there were no significant differences in concentrations between the commercial and standard formulations and between the isolated and mixed applications, except for the ternary mixture (D + H + S) at D5 (20–25 cm), which was superior to the other treatments in the commercial formulation.

The low solubility of diuron and its greater affinity for hydrophobic compounds might have contributed to its retention in the most superficial layers of the soil, resulting in low leaching in the soil profile [26,27]. In addition, LV is a highly weathered soil with a higher clay content, favoring the occurrence of adsorption processes, such as montmorillonite (Figure 1). Montmorillonite is an expansive clay mineral with a large internal surface area due to its interlayer spacing, which provides considerable space for the storage of organic compounds [28]. Studies have indicated that expansive clay minerals act as excellent natural adsorbents in sediments due to their active structural sites, such as exchangeable cations, hydrophobic surfaces, and Si-OH [29]. Other studies have reported that the isolated application of the analytical standard diuron remained most concentrated in the top 15 cm of both sandy and clayey soil depths [30,31], which is consistent with our findings.

#### 3.1.2. Haplic Cambisol (CX)

**Figure 5:**
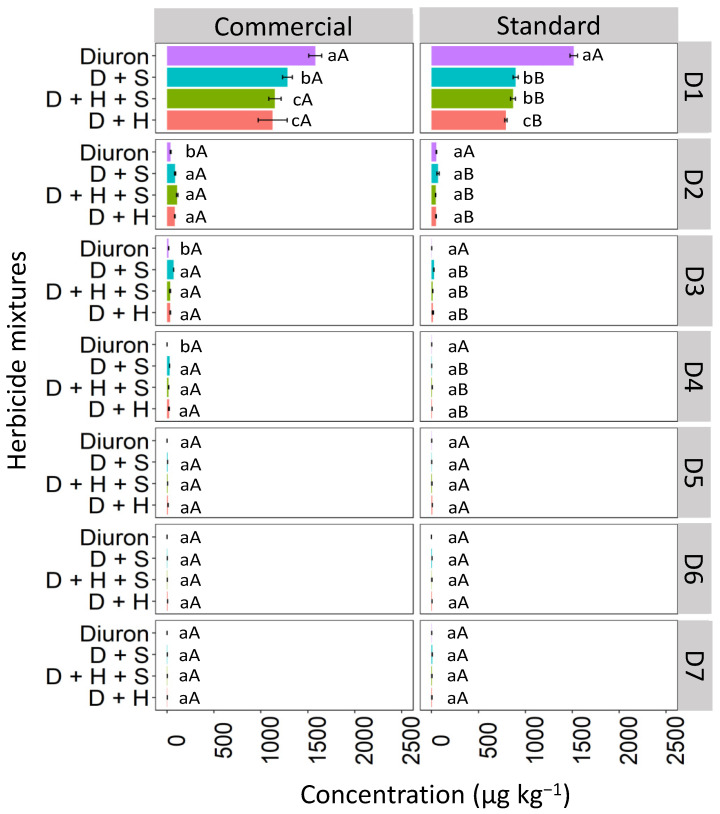
Leaching (μg kg^−1^) of diuron isolated and in binary and ternary mixtures for commercial and standard formulation in the Haplic Cambisol. Lowercase letters compare means between mixtures, and uppercase letters compare formulations (commercial and standard). Bars indicate the standard error of the mean (n = 3). D + S (diuron + hexazinone), D + H + S (diuron + hexazinone + sulfometuron-methyl), D + H (diuron + hexazinone). D1 to D7 represent the soil depths.

Diuron leaching in Haplic Cambisol (CX) was affected by the binary and ternary mixtures, both in the commercial and standard formulations (Figure 5). In the commercial formulation of D1, the diuron isolated had the highest concentration (1611.0 μg kg^−1^), followed by the D + S mixture (1239.1 μg kg^−1^) and the D + H + S and D + H mixtures with 1150.1 and 1099.2 μg kg^−1^, respectively. In the standard formulation, the diuron isolated had the highest concentration (1500.1 μg kg^−1^), followed by the mixtures D + S (912.4 μg kg^−1^), D + H + S (850.5 μg kg^−1^), and finally the D + H mixture, which presented 627.3 μg kg ^−1^, which corresponds to a reduction of 58.18% compared to isolated diuron. At D2, there was no statistical difference between isolated diuron in commercial and standard formulations. However, when comparing the mixtures, it was observed that in the commercial formulation, diuron leached more prominently into the D2 layer compared to the standard formulation. Similar behavior was observed at depths D3 and D4, indicating that diuron tends to leach more in the commercial formulation, especially when applied as binary and ternary mixtures. From depth D5 to D7, diuron was no longer detected, whether applied in isolation or as mixtures, for both types of formulations.

The higher concentration of isolated diuron in the most superficial layer, compared to applications in mixtures, may be related to competition for the sorting sites available in the CX. Furthermore, when composts are sorbed, transport and biodegradation processes become slower, leading to compost accumulation in the upper layers of the soil, reducing the risk of leaching [32]. Herbicides that contain three nitrogen molecules with heterocyclic aromatic rings are more competitive than herbicides with a substituted benzene ring in the structure and are therefore preferentially absorbed first [33]. This may be one reason that explains why the diuron present in the mixtures is less adsorbed to soil colloids than its isolated application, as its molecule has a substituted benzene ring, making it less competitive than hexazinone, which has a heterocyclic aromatic ring with three nitrogen atoms.

#### 3.1.3. Quartzarenic Neosol (RQ)

**Figure 6:**
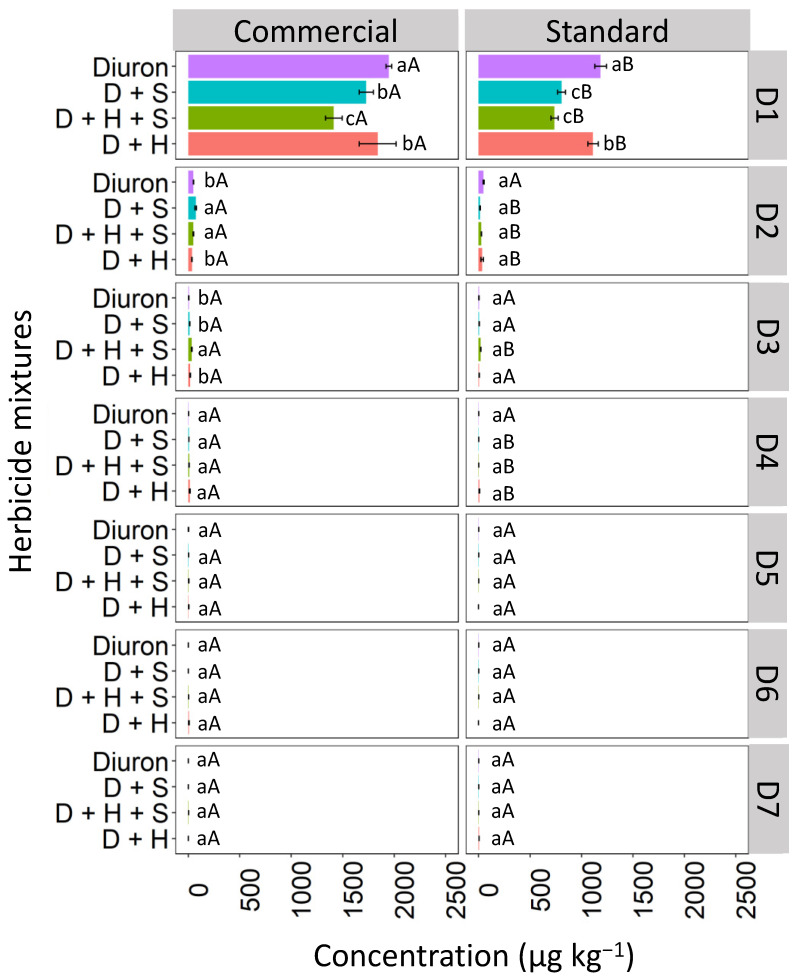
Leaching (μg kg^−1^) of diuron isolated and in binary and ternary mixtures for commercial and standard formulation in Quartzarenic Neosol. Lowercase letters compare means between mixtures, and uppercase letters compare formulations (commercial and standard). Bars indicate the standard error of the mean (n = 3). D + S (diuron + hexazinone), D + H + S (diuron + hexazinone + sulfometuron-methyl), D + H (diuron + hexazinone). D1 to D7 represent the soil depths.

The highest concentration of diuron, whether in isolation or in binary and ternary mixtures, was observed in the commercial formulation compared to the standard formulation (Figure 6). At D1, a higher concentration of diuron isolated and in mixtures was observed in the commercial formulation compared to the standard formulation. In the commercial formulation, the concentration of isolated diuron (1958.3 μg kg^−1^) was the highest, followed by D + H (1811.5 μg kg^−1^), D + S (1723.6 μg kg^−1^), and D + H + S (1415.7 μg kg^−1^). Conversely, in the standard formulation, at D1, isolated diuron (1243.7 μg kg^−1^) predominated, followed by the D + H mixture (1136.8 μg kg^−1^), D + S (853.2 μg kg^−1^), and D + H + S (761.4 μg kg^−1^). At D2, the concentration of isolated diuron exhibited similar behavior in both commercial and standard formulations. In the mixtures, it was found that the commercial formulation had higher concentrations than the standard formulation. In the commercial formulation, the herbicides D + S and D + H + S were more leached into D2 than the isolated diuron and the D + H mixture. For the standard formulation, all concentrations of diuron at D2, both applied in isolated form and mixtures, had the same behavior. At D3, only the ternary mixture of the commercial formulation showed a higher concentration than the standard formulation, and the other herbicides applied had similar leaching concentration. The isolated diuron had the same behavior between the commercial and standard formulations at D4, while the commercial formulation mixtures had higher concentrations than the standard formulation. At other depths (D5–D7), the presence of diuron isolated and in mixtures in commercial and standard formulations was not detected.

The low leaching of diuron in the RQ soil might be due to the high organic matter content present in the soil (12.05 g kg^−1^), since RQ soils have a low capacity to retain water and nutrients [34]. Some studies have shown a strong correlation between diuron retention and soil organic matter content [35]. Furthermore, the high hydrophobicity of diuron increases its affinity for lipophilic adsorption sites on organic matter [36]. This fact occurs because organic matter favors hydrogen bonds due to carboxylic and phenolic groups, increasing the adsorption stability of the herbicide [37].

### 3.2. Leaching of Commercial Formulations and Hexazinone Analytical Standard

#### 3.2.1. Red Latosol (LV)

**Figure 7:**
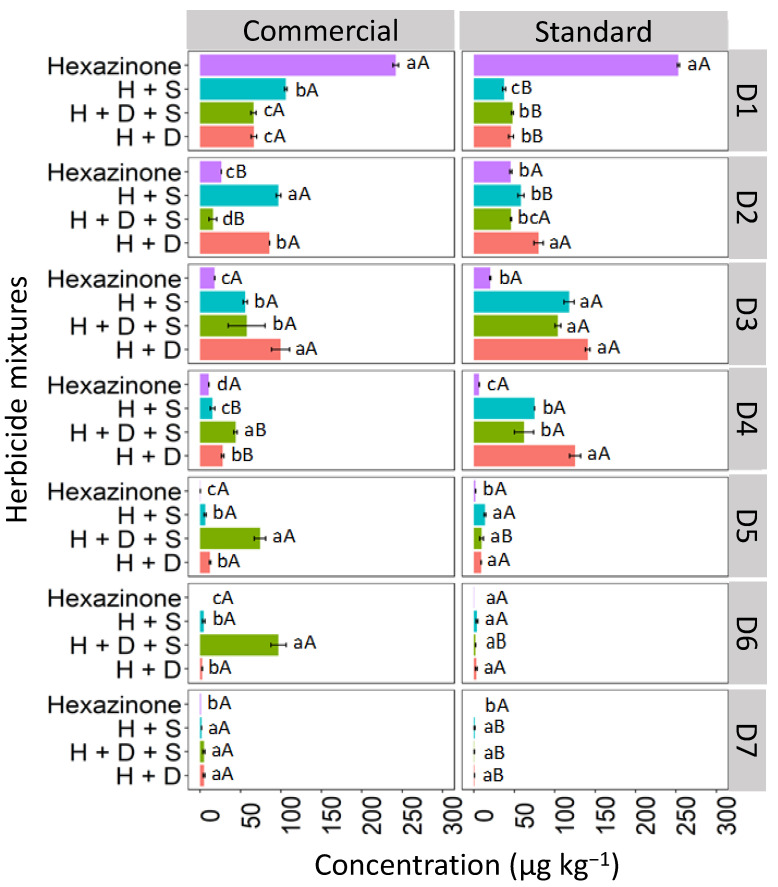
Leaching (μg kg^−1^) of hexazinone, isolated and in binary and ternary mixtures, for commercial and standard formulation in Red Latosol. Lowercase letters compare means between mixtures, and uppercase letters compare formulations (commercial and standard). Bars indicate the standard error of the mean (n = 3). H + S (hexazinone + sulfometuron-methyl), H + D + S (hexazinone + diuron + sulfometuron-methyl), H + D (hexazinone + diuron). D1 to D7 represent the soil depths.

The isolated hexazinone was leached up to D4, verifying a similar behavior in depths D1, D3, and D4 for the two studied formulations (commercial and standard; Figure 7). At D2, the isolated hexazinone had a higher concentration in the standard formulation (42.7 μg kg^−1^) compared to the commercial one (26.4 μg kg^−1^). For hexazinone applications in the mixture, leaching was observed up to D7 for both the commercial and standard formulations, with the ternary mixture exhibiting notable concentrations at depths D5 (68.2 μg kg^−1^) and D6 (97.8 μg kg^−1^) in the commercial formulation. This suggests that when hexazinone is used in a ternary mixture with diuron and sulfometuron-methyl, it exhibits greater leaching in LV soil compared to its isolated application. The high solubility of hexazinone in water (33,000 mg L^−1^ at 25 °C) increases the risk of leaching of this herbicide [38]. Furthermore, the pH of the LV soil (4.7), as used in this study, was higher than the pKa value of hexazinone (2.2). As a result, the herbicide existed predominantly in its molecular form in the soil solution, which favored its leaching. The presence of diuron and sulfometuron-methyl increased hexazinone leaching. These herbicides may have competed for soil sorption sites, leaving hexazinone more available in solution and more prone to leaching. Mendes et al. [27], evaluating the leaching of hexazinone mixed with diuron and sulfometuron-methyl in soils with contrasting textures, found a higher percentage recovery of hexazinone relative to the amount applied in the 0–0.20 m layer in sandy clay-texture soil and 0.20–0.30 m in sandy sand-textured soil, showing the high mobility of this herbicide in the soil profile.

#### 3.2.2. Haplic Cambisol (CX)

**Figure 8:**
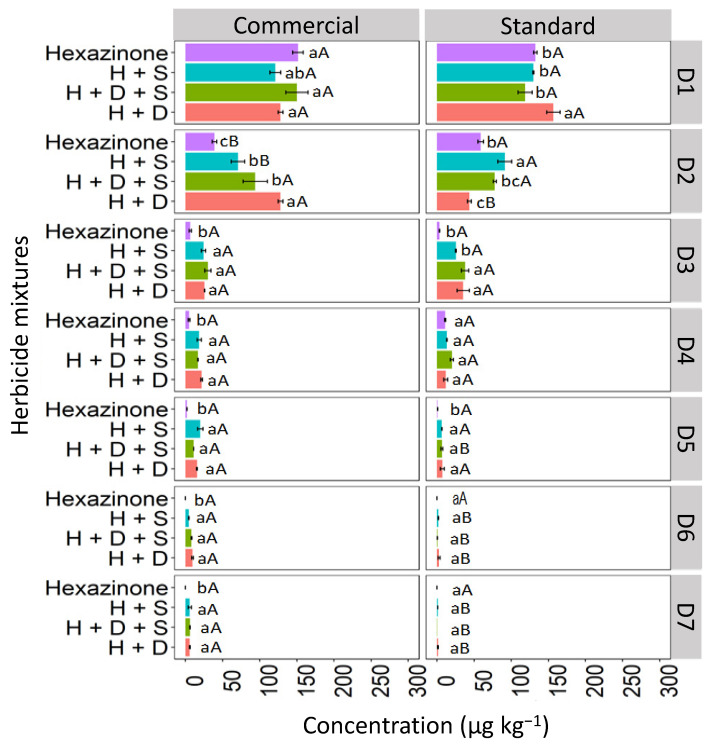
Leaching (μg kg^−1^) of hexazinone isolated and in binary and ternary mixtures for commercial and standard formulation in the Haplic Cambisol. Lowercase letters compare means between mixtures, and uppercase letters compare formulations (commercial and standard). Bars indicate the standard error of the mean (n = 3). H + S (hexazinone + sulfometuron-methyl), H + D + S (hexazinone + diuron + sulfometuron-methyl), H + D (hexazinone + diuron). D1 to D7 represent the soil depths.

In the CX soil, at depth D1 (0–5 cm), all concentrations of hexazinone applied in isolated form and mixtures exhibited similar behavior for both the commercial and standard formulations (Figure 8). However, at D2 (5–10 cm), isolated hexazinone displayed distinct behavior between the two formulations, with a reduction in concentration in the commercial formulation (28.7 μg kg^−1^) compared to the standard formulation (63.4 μg kg^−1^). This reduction was detected up to D5 (20–25 cm) for both formulations. Binary mixtures (H + S and H + D) and the ternary mixture (D + H + S) were detected as deep as D7 (30–35 cm) in both formulations, with more pronounced concentrations of the mixtures observed at D6 (25–30 cm) and D7 (30–35 cm) in the commercial formulation.

These leaching results are similar to the findings reported by da Silva et al. [39] for Cambisol, Ferrasol, and Arenosol, where the standard formulation of hexazinone was quantified up to a depth of D5 (0.20–0.25 cm). In our study, the higher leaching observed in the CX for the commercial formulation compared to the standard formulation is probably a result of the insertion of additives in the commercial formulation to increase the active ingredient in the soil solution, thus facilitating the absorption by plants. It was reported that product additives, including solvents, surfactants, spreaders, and adhesives, can be included as co-formulants to improve the performance of an active substance by modifying the physical characteristics and particles of the spray mixture [1]. Working with two soils from York, United Kingdom, these same authors found more significant leaching of propyzamide using the commercial than the standard formulation. Other authors reported that alkyl polyglucoside (APG) surfactants used as foaming agents increased the mobility of diuron and glyphosate in leach columns mounted with washed sand [40].

#### 3.2.3. Quartzarenic Neosol (RQ)

**Figure 9:**
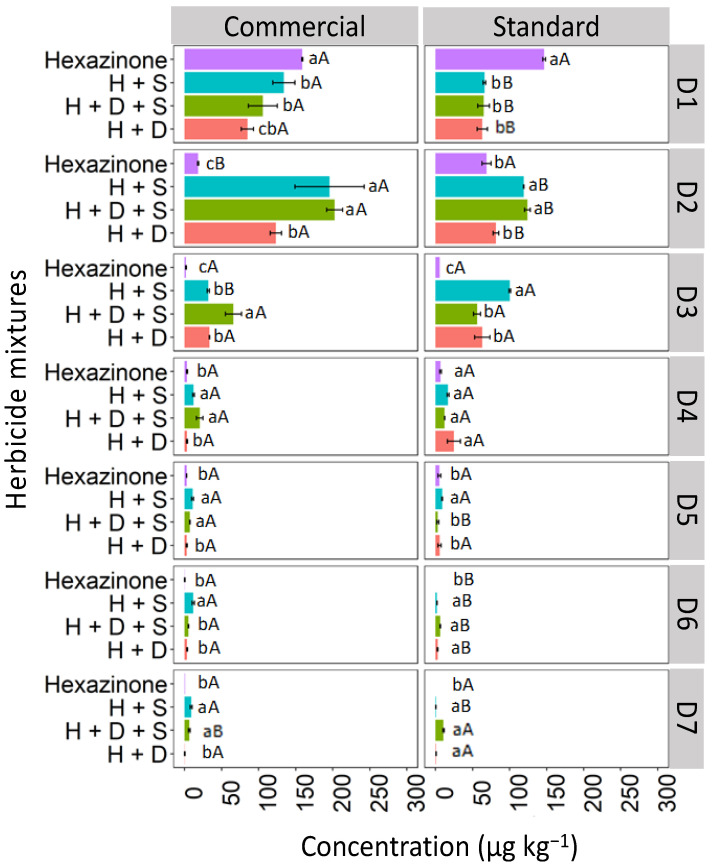
Leaching (μg kg^−1^) of hexazinone isolated and in binary and ternary mixtures for commercial and standard formulation in Quartzarenic Neosol. Lowercase letters compare means between mixtures, and uppercase letters compare formulations (commercial and standard). Bars indicate the standard error of the mean (n = 3). H + S (hexazinone + sulfometuron-methyl), H + D + S (hexazinone + diuron + sulfometuron-methyl), H + D (hexazinone + diuron). D1 to D7 represent the soil depths.

The leaching of hexazinone isolated and in binary and ternary mixtures in the Quartzarenic Neosol (RQ) is presented in Figure 9. The highest concentration of isolated hexazinone was observed at D1 (158.9 and 150.1 μg kg^−1^) for the commercial and standard formulations, respectively. The concentrations were quantified at lower levels up to D6 for the commercial formulation and up to D5 for the standard formulation. However, when hexazinone was applied in mixtures (binary and ternary), the concentrations remained relatively uniform up to depth D3 (10–15 cm), decreasing as you go deeper, up to D7 (30–35 cm), for both the commercial and standard formulations. Although hexazinone was quantified up to D7 in the soil, its presence was not detected in the leachate of any of the evaluated treatments.

The higher leaching observed for hexazinone when mixed compared to when isolated in RQ can be explained by the fact that mixed herbicide molecules tend to interact with each other through hydrophobic or π–π interactions, resulting in greater competition for binding sites, becoming more available in the soil solution, with a consequent increase in leaching. This explanation agrees with El-Nahhal and Hamdona [41], who found that herbicide molecules in the mixture can form a larger organic molecule that reacts with soil organic matter or the clay fraction, forming an organoclay complex that can strongly adsorb the herbicide molecules and release them slowly into the soil environment, thereby minimizing their phytotoxic effects.

### 3.3. Leaching of Commercial Formulations and the Analytical Standard of Sulfometuron-Methyl

#### 3.3.1. Red Latosol (LV)

**Figure 10:**
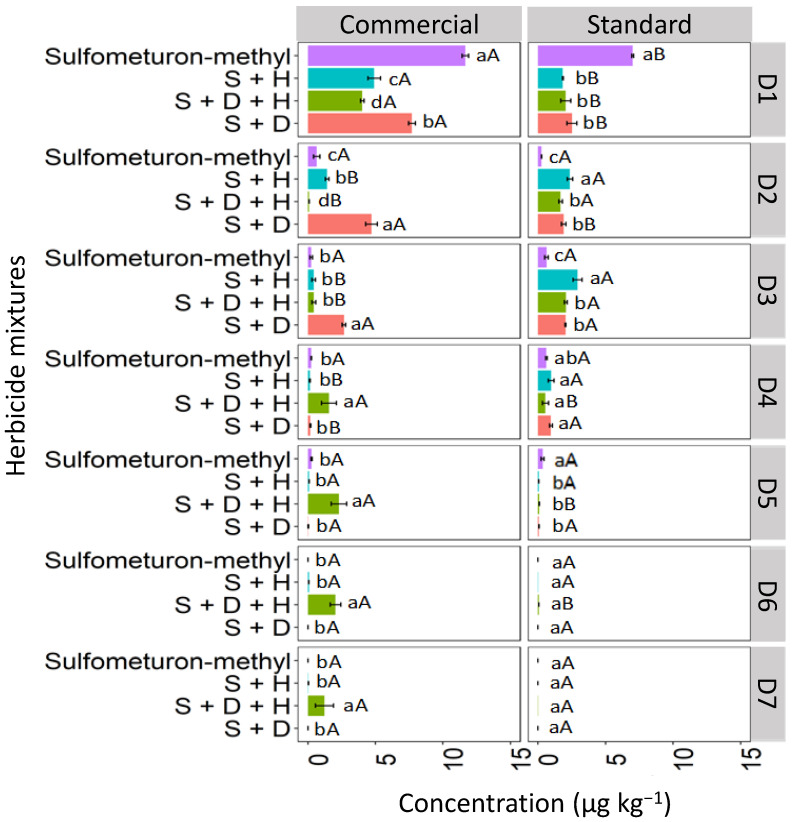
Leaching (μg kg^−1^) of sulfometuron-methyl isolated and binary and ternary mixtures for commercial and standard formulation in Red Latosol. S + H (sulfometuron-methyl + hexazinone), S + D + H (sulfometuron-methyl + diuron + hexazinone), S + D (sulfometuron-methyl + diuron). Lowercase letters compare means between mixtures, and uppercase letters compare formulations (commercial and standard). Bars indicate the standard error of the mean (n = 3). D1 to D7 represent the soil depths.

Binary and ternary mixtures used in different formulations (commercial and standard) affected sulfometuron-methyl leaching compared to sulfometuron-methyl applied isolated in the LV soil (Figure 10). The highest concentration of isolated sulfometuron-methyl was detected at D1 (0–5 cm), with 12.3 μg kg^−1^ for the commercial formulation and 7.7 μg kg^−1^ for the standard formulation. Sulfometuron-methyl leached at lower concentrations until D5 (20–25 cm). The binary mixture S + H was quantified significantly at D1 for the commercial formulation (4.9 μg kg^−1^). In contrast, in the standard formulation, the highest concentration of this mixture was verified at D3 (10–15 cm; 2.5 μg kg^−1^), revealing more significant leaching in this formulation. D + H + S reached the highest depths for the commercial formulation, reaching a concentration of 1.2 μg kg^−1^ at D7 (30–35 cm). The ternary standard formulation was detected up to D4 (15–20 cm). The binary mixture S + D had the highest concentration at D1 (7.5 μg kg^−1^) for the commercial formulation and was leached in smaller concentrations up to D4 (15–20 cm) for both formulations.

The increased leaching of sulfometuron-methyl observed in the ternary mixture, especially in the commercial formulation, may be attributed to the greater competitive sorption among the three herbicide molecules, leading to more pronounced leaching. Studies suggest that the slow separation of the pesticide molecule from the additives present in the commercial formulation surrounding the soil can decrease the rate of sorption processes about the technical material [42]. Furthermore, other researchers associate that the presence of additives may favor the maintenance of pesticide molecules in solution, weakening the sorption to that of technical pesticide material [43].

#### 3.3.2. Haplic Cambisol (CX)

**Figure 11:**
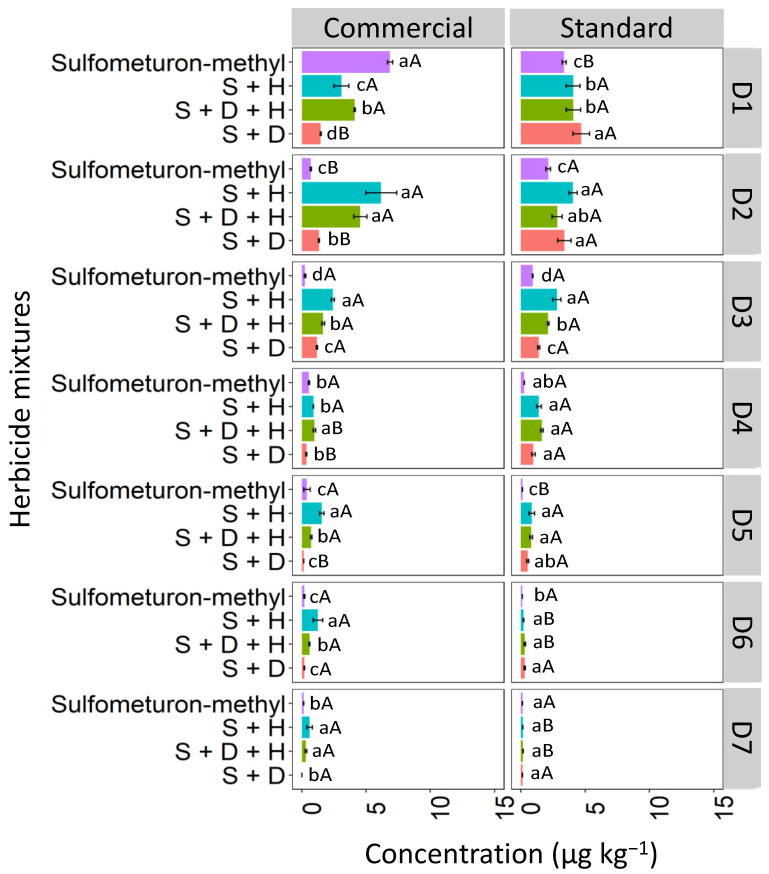
Leaching (μg kg^−1^) of sulfometuron-methyl isolated and in binary and ternary mixtures for commercial and standard formulation in the Haplic Cambisol. S + H (sulfometuron-methyl + hexazinone), S + D + H (sulfometuron-methyl + diuron + hexazinone), S + D (sulfometuron-methyl + diuron). Lowercase letters compare means between mixtures, and uppercase letters compare formulations (commercial and standard). Bars indicate the standard error of the mean (n = 3). D1 to D7 represent the soil depths.

In the CX soil, at D1, the isolated commercial formulation of sulfometuron-methyl exhibited the highest concentration compared to the sulfometuron-methyl in mixtures, reaching a concentration of 7.4 μg kg^−1^ (Figure 11). At D1, for the standard formulation, the binary mixture S + D showed the highest concentration (5.7 μg kg^−1^). At D2, the highest concentrations of sulfometuron-methyl observed in the commercial formulation were those applied in the mixtures S + H (6.2 μg kg^−1^) and S + D + H (4.8 μg kg^−1^). In the standard formulation, the highest concentrations were observed in the mixtures S + H (3.9 μg kg^−1^), S + D + H (2.8 μg kg^−1^), and S + D (3.4 μg kg^−1^). At D3, the concentrations of sulfometuron-methyl showed similar behavior both within and between the commercial and standard formulations, with the order of concentration being S + H > S + D + H > S + D > S. Sulfometuron-methyl, both isolated and in mixtures, was detected up to D7 in the two formulations; however, it was observed that the mixtures containing hexazinone (D + H and D + H + S) concentrations were more accentuated, especially in the commercial formulation.

Sulfometuron-methyl is a weak acid with moderate solubility in water (300 mg L^−1^ at pH 7–5 °C), with an acid dissociation constant (pKa) of 5.2, which justifies the high mobility of sulfometuron-methyl observed in CX for the two studied formulations (commercial and standard). In these conditions, with the soil pH at 7.6, which is higher than the herbicide pKa (5.2), the molecules exist primarily in their dissociated form. This makes them more readily available in the soil solution, making leaching more likely and favorable. Studies evaluating the sorption, desorption, and leaching potential of sulfonylurea herbicides in Argentine soils found that the sorption coefficients were correlated with pH and organic carbon content. Regardless of factors such as landscape position, soil depth, and decomposition rate in surface soils, sulfometuron-methyl has been classified as a leachable herbicide [44]. Silva et al. [8] evaluated the sorption and desorption coefficients of diuron, hexazinone, and sulfometuron-methyl in 15 soils from different Brazilian states, and reported that these herbicides have a high potential risk for groundwater contamination.

#### 3.3.3. Quartzarenic Neosol (RQ)

**Figure 12:**
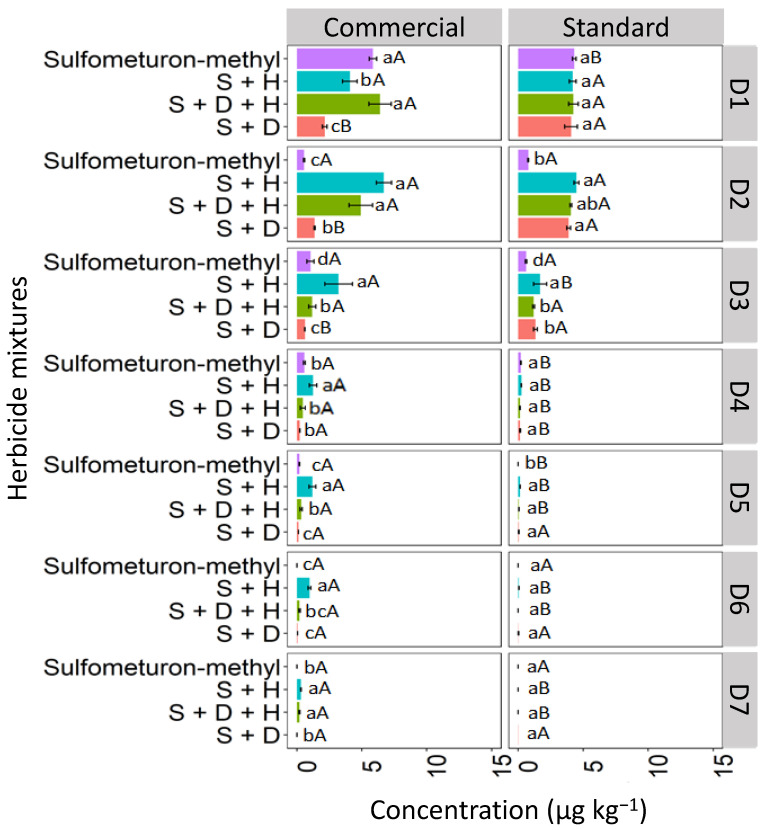
Leaching (μg kg^−1^) of sulfometuron-methyl, isolated and binary and in ternary mixtures, for commercial and standard formulation in Neosol. S + H (sulfometuron-methyl + hexazinone), S + D + H (sulfometuron-methyl + diuron + hexazinone), S + D (sulfometuron-methyl + diuron). Lowercase letters compare means between mixtures, and uppercase letters compare formulations (commercial and standard). Bars indicate the standard error of the mean (n = 3). D1 to D7 represent the soil depths.

The concentration of isolated sulfometuron-methyl quantified at D1 was higher in the commercial formulation, with 6.7 μg kg^−1^, than in the standard formulation, which was 4.1 μg kg^−1^ in the RQ soil (Figure 12). In the commercial formulation, the isolated sulfometuron-methyl and the ternary mixture (S + D + H) presented higher concentrations at D1 than the binary mixtures S + H and S + D. In the standard mixture, the isolated sulfometuron-methyl and mixtures presented similar behavior at D1. At D2, the isolated sulfometuron-methyl had a lower concentration compared to the mixtures in the commercial and standard formulations. In the commercial formulation, S + H (7.1 μg kg^−1^) and S + D + H (4.7 μg kg^−1^) presented higher concentrations. The binary mixture S + H exhibited higher concentrations from D3 to D7 compared to the isolated application and the mixtures S + D + H and S + D in the commercial formulation. This behavior set it apart from the standard formulation, which could only quantify sulfometuron-methyl from the mixture S + H up to D5. This demonstrates that the leaching of S + H was more pronounced in the commercial formulation.

These results align with findings by Földényi et al. [13], who reported a significant decrease in the adsorption of chlorsulfuron in sandy soil when the forming agent Supragil was present. This suggests that the leaching of sulfometuron-methyl in RQ was likely influenced by the soil’s pH (4.9), since, with a pKa 5.2 higher than the pH, the molecular form predominates in the solution, favoring the sorption process. This phenomenon was more pronounced in the standard formulation, where sulfometuron-methyl exhibited lower leaching.

### 3.4. Implications of the Results on Weed Control, Potential Environmental Impact, and Future Studies

In general, the results obtained show essential differences between the behavior of herbicide molecules in the commercial formulation and the analytical standard. This fact shows that research already carried out on the herbicide transport process using an analytical standard may have underestimated the transport of molecules in the soil profile. In addition, the leaching intensity seems to be more remarkable for herbicides considered mobile, such as hexazinone and sulfometuron-methyl, compared to diuron, notably of low mobility [8,27].

The higher concentration of diuron in the topsoil layer is not a new finding [29,45,46]. Nevertheless, when used in a mixture, more significant leaching can be advantageous for weed control, as it results in a greater concentration of the herbicide at greater soil depths, allowing for better control of germinating weeds. This increased transport of diuron does not appear to pose environmental concerns due to its low concentration and its presence being limited to the upper soil layers. However, it is important to note that this behavior may be influenced by rainfall volume [30,47,48] and should be taken into consideration.

The greater mobility of hexazinone and sulfometuron-methyl in relation to diuron in the soils used in this study was an expected behavior and demonstrates the potential of herbicides to control weed propagules present in the soil [5]. However, the more significant leaching of these herbicides when in mixtures raises concerns, as it results in a greater quantity of products outside the location zone of the weed propagules [49]. In addition, this increased transport can generate the most significant contamination, mainly of groundwater [50,51].

The present research also demonstrated that studies on the environmental fate of herbicides based only on the interactions between standard formulations (analytical grade) and the environment, ignoring the effects of existing additives in commercial formulations, may be hiding the reality that occurs in agricultural fields. Therefore, the authors encourage that more research on the subject be carried out in order to elucidate the interactions that can occur due to the mixing of products in tanks or formulated products, as a way to assist crop advisor professionals to make safer recommendations for the use of herbicides in agricultural fields.

## 4. Conclusions

The leaching of diuron, hexazinone, and sulfometuron-methyl herbicides in soils is higher in the commercial formulation than the analytical standard. The mixture of herbicides alters the transport potential in soils, being more intense for hexazinone and sulfometuron-methyl. This increased transport, resulting from mixing and commercial formulation, can enhance the availability of herbicides in soils. Consequently, it has the potential to alter the effectiveness of weed control and contribute to the contamination of subsurface water resources.

## Figures and Tables

**Table 1:** Physical and chemical characterization of the three soils used in the experiments, Mossoró-RN, Brazil.

Attributes	Soils		
	LV	CX	RQ
Texture	Sandy loam	Sandy clay	Loamy sand
Sand (g kg^−1^)	810.00	500.00	840.00
Silt (g kg^−1^)	50.00	150.00	100.00
Clay (g kg^−1^)	140.00	350.00	60.00
pH (water)	4.70	7.60	4.90
OM (g kg^−1^)	9.24	14.06	12.05
P (mg dm^−3^)	5.10	4.50	4.30
K (mg dm^−3^)	55.50	574.50	95.20
Ca^2+^ (cmol_c_ dm^−3^)	1.10	11.70	1.40
Mg^2+^ (cmol_c_ dm^−3^)	0.20	1.00	0.50
Al^3+^ (cmol_c_ dm^−3^)	0.10	0.00	0.15
H + Al (cmol_c_ dm^−3^)	1.65	0.00	1.65
CEC (cmol_c_ dm^−3^)	3.16	14.29	3.99
V (%)	48.00	100.00	50.00
m (%)	6.00	0.00	6.00

Haplic Cambisol (CX), Red Latosol (LV), Quartzarenic Neosol (RQ). Hydrogenic potential (pH), organic matter (OM), phosphorus (P), sodium (Na^+^), potassium (K), calcium (Ca^2+^), magnesium (Mg^2+^), aluminum (Al^3+^), potential acidity (H + Al), cation exchange capacity (CEC), base saturation (V), aluminum saturation (m). The analyses were carried out according to the methodology proposed by the Brazilian Agricultural Research Corporation EMBRAPA [17]. Soil, Water, and Plant Analysis Laboratory (LASAP) at UFERSA.

**Table 2:** Validation parameters of the analytical method by UHPLC.

Herbicide	Linearity		Matrix Effect	Repeatability	LOD	LOQ
	Interval µg kg^−1^	R^2^	(%)	RSD (%)	µg kg^−1^	µg kg^−1^
Diuron	0.5–50	0.9998	13.38	1.74–6.67	0.91	2.76
Hexazinone	0.5–50	0.9980	5.57	1.22–3.69	1.28	3.91
Sulfometuron-methyl	0.5–50	0.9996	−3.59	1.46–5.50	0.81	2.46

Limit of detection (LOD); limit of quantification (LoQ); relative standard deviation (RSD); R^2^, coefficient of determination.

**Table 3:** Percentage recovery for three concentration levels for diuron, hexazinone, and sulfometuron-methyl.

Herbicide	Concentration					
	1 µg kg^−1^		10 µg kg^−1^		100 µg kg^−1^	
	Recovery (%)	RSD (%)	Recovery (%)	RSD (%)	Recovery (%)	RSD (%)
Diuron	109.87	3.25	100.33	2.28	106.16	6.36
Hexazinone	106.32	2.48	94.69	7.31	96.78	3.64
Sulfometuron-methyl	81.37	1.24	87.01	7.59	84.15	3.05

Relative standard deviation (RSD).

**Table 4:** Multiple reaction monitoring (MRM) transitions and optimized parameters.

Herbicide	Retention Time (min^−1^)	Quantification			Confirmation		
		MRM ^a^ Transition *m*/*z*	DP ^b^ (V)	CE ^c^ (V)	MRM ^a^ Transition *m*/*z*	DP ^b^ (V)	CE ^c^ (V)
Diuron	2.51	233.0 ˃ 72.0	28	19	233.0 ˃ 160.0	29	25
Hexazinone	2.10	253.1 ˃ 70.3	29	54	253.1 ˃ 170.5	16	30
Sulfometuron-metyl	2.18	365 ˃ 150.0	28	17	365.0 ˃ 106.8	19	45

^a^ Multiple reaction monitoring; ^b^ decomposition potential; ^c^ collision energy.

## Data Availability

Data are available upon request.
